# Function of Serum Complement in Drinking Water Arsenic Toxicity

**DOI:** 10.1155/2012/302817

**Published:** 2012-03-21

**Authors:** Laila N. Islam, M. Shamim Hasan Zahid, A. H. M. Nurun Nabi, Mahmud Hossain

**Affiliations:** Department of Biochemistry and Molecular Biology, University of Dhaka, Dhaka 1000, Bangladesh

## Abstract

Serum complement function was evaluated in 125 affected subjects suffering from drinking water arsenic toxicity. Their mean duration of exposure was 7.4 ± 5.3 yrs, and the levels of arsenic in drinking water and urine samples were 216 ± 211 and 223 ± 302 *μ*g/L, respectively. The mean bactericidal activity of complement from the arsenic patients was 92% and that in the unexposed controls was 99% (*P* < 0.01), but heat-inactivated serum showed slightly elevated activity than in controls. In patients, the mean complement C3 was 1.56 g/L, and C4 was 0.29 g/L compared to 1.68 g/L and 0.25 g/L, respectively, in the controls. The mean IgG in the arsenic patients was 24.3 g/L that was highly significantly elevated (*P* < 0.001). Arsenic patients showed a significant direct correlation between C3 and bactericidal activity (*P* = 0.014). Elevated levels of C4 indicated underutilization and possibly impaired activity of the classical complement pathway. We conclude reduced function of serum complement in drinking water arsenic toxicity.

## 1. Introduction

Drinking water contamination with high levels arsenic in Bangladesh is the largest mass poisoning of a population in history, with millions of people exposed [1]. The mean arsenic concentrations in the drinking water of the exposed populations in Bangladesh are more than twenty times higher [2] than the maximum permissible limit recommended by the Environmental Protection Agency of the USA that has set the level at 10 micrograms per liter or 10 parts per billion. A total of 61 out of 64 districts in Bangladesh are already affected with high levels of arsenic in ground water. We found melanosis with black/white pigmentation on skin, keratosis, lump, and nodule formation on feet and hands in people chronically exposed to high levels of arsenic in drinking water [2]. It had been reported that symptoms of chronic arsenic toxicity develop insidiously after 6 months to 2 years or more of exposure [3].

The general adverse health effects associated with human exposure to arsenicals include cardiovascular diseases, developmental abnormalities, neurologic and neurobehavioral disorders, diabetes, fibrosis of the liver and lung, and haematological disorders [4]. We reported, that apart from the classical arsenical skin lesions, a large proportion of arsenicosis patients had breathing problems, gastric and abdominal pain, backache, headache, pain all over the body, palpitation, anemia, and weakness. Arsenic toxicity caused respiratory complications [5], modulation of serum metabolites [6], and affected the immune system with low leukocyte count [2], and elevated levels of serum immunoglobulins [5]. However, the functions of serum complements have not been studied in the arsenicosis patients suffering from such a wide range of complications.

The complement system plays an important role in defense against pyogenic infections. Complements are innate components of the immune system whose activation leads to robust and efficient proteolytic cascades, which terminate in opsonization and lysis of the pathogen as well as in the generation of the classical inflammatory response through the production of potent proinflammatory molecules [7]. Complement activation also participates in clearance of apoptotic cells and immune complexes. Recently, it was also recognized that complement plays a key role in adaptive immunity by modulating and modifying the T-cell responses [8]. Therefore, the complement systems may be regarded as a link between innate and adaptive immunity and is critically involved in the pathogenesis and prevention of immune complex diseases, such as systemic lupus erythematosus (SLE) [9].

The immunotoxicity of heavy metals has not been extensively studied. Bernier et al. [10] found that heavy metals including mercury, lead, and cadmium present in small amounts in the Great Lakes water and fish and exposure of humans to these metals via the ingestion of contaminated food especially fish alter a number of parameters of the host's immune system leading to increased susceptibility to infections, autoimmune diseases, and allergic manifestations. In another study, it was found that mercury can induce autoimmune disease both in humans and experimental animals, while cadmium treatment of rats and mice results in autoimmune responses that vary with species and inbred strain of animals [11]. The peripheral blood neutrophils of workers occupationally exposed to lead showed reduced chemotaxis and nitroblue tetrazolium dye reduction, suggesting immune dysfunction may be a sensitive indicator of lead exposure [12]. In view of lack of information on the humoral immune response, this study was designed to evaluate serum complement function in arsenicosis patients.

## 2. Materials and Methods

### 2.1. Study Area

The study areas of this investigation were the arsenic endemic rural villages of Rajarampur, Achinpara, Chandnai, and Bottola under the northwestern district of Chapainawabganj where arsenic contamination in drinking water was first detected in Bangladesh in 1993 and the village Ilumdi under Araihazar of Narayanganj that is about 35 km southeast of the capital city Dhaka.

### 2.2. Arsenicosis Patient Inclusion Criteria

Subjects consuming drinking water that contained arsenic levels greater than the tolerable limit recommended by the WHO (50 *μ*g/L), not taking medicine for any illness, and having signs of arsenic toxicity were enrolled in this study as the patients. Their source of drinking water was tube well or artisan well. The signs of arsenic toxicity (arsenicosis) were assessed on the basis of appearance of skin lesions [2, 5, 6]. After informed consent, the researchers with the help of a public health nurse interviewed all the patients personally. The information on symptoms and complications of arsenic toxicity, duration of exposure, anthropometric parameters including age, height, and body weight of the patients were recorded on preformed questionnaires.

### 2.3. Sample Collection

Blood samples (about 3 mL) were collected from the subjects with their full consent to participate in this study. A total of 250 blood samples were collected of which 125 were from patients with arsenic toxicity (arsenicosis), 95 from unexposed subjects living in the same area as the patients but drinking safe water and with no sign of arsenic toxicity, and the remaining from unexposed subjects living in the city. Spot urine and drinking water samples were also collected from each individual. All samples were collected in fresh, sterile universals. The blood sample was allowed to clot, and serum was separated immediately after collection. The frozen sera were transported to the laboratory in ice-packed containers and stored at −80°C until analyzed.

### 2.4. Analyses of Arsenic

Concentrated nitric acid was mixed with the drinking water samples at 1 mL per liter and stored at 4°C until analyzed. The urine samples were frozen immediately after collection. Arsenic content in the water and urine samples were measured following the standard procedure by using a Flow Injection—Hydride Generation—Atomic Absorption Spectrophotometer, and the results were expressed in *μ*g/L.

### 2.5. Assay of Complement-Mediated Bactericidal Activity

*Escherichia coli* DH5*α* was grown in nutrient broth for 14 hour at 37°C in an orbital shaker. The bacterial cells were harvested, washed two times using excess of PBS and then the suspension was adjusted to 0.600 O.D. at 620 nm. Immediately, aliquots of 200 *μ*L of the bacterial cell suspensions (BCS) were taken into separate tubes, 20 *μ*L of serum was added to each tube, and the mixture was incubated for 30 minutes at 37°C. At the end of incubation, the remaining viable cells were serially diluted with PBS to 1 : 10,000. An aliquot of 20 *μ*L of this dilution was spread on each of 3 agar plates and incubated for 16 hours at 37°C. The number of colonies formed was counted, and the mean value for each serum was taken from the readings of 3 plates. For the negative control experiments, 20 *μ*L of PBS (medium) was added to the BCS instead of serum, incubated, and then serially diluted with PBS to 1 : 50,000. An aliquot of 20 *μ*L of this dilution was spread on each of 3 agar plates.

### 2.6. Assay of Complement-Inactivated Bactericidal Activity

Complement proteins were inactivated by heat treatment at 56°C for 30 minutes in a water bath and then used (20 *μ*L) to test for bactericidal activity. Both the PBS (control) and bacteria treated with inactivated serum preparations were serially diluted to 1 : 50,000. The rest of the procedure was as described before.

### 2.7. Calculation of Bactericidal Activity

Bactericidal activity was calculated using the following formula, as described elsewhere [13, 14]. For the control, if the mean colony-forming unit (cfu) on the plate was Nc, then 1 mL of the original bacterial cell suspension contained Nc × 50 × 50,000 cfu.

For the test serum, if the mean cfu on the plate was Ns, then 1 mL of the bacterial cell suspension treated with serum complement contained Ns × 50 × 10,000 cfu. Therefore,

(1) $$\begin{array}{cc} & \%\text{bactericidal}\;\text{activity}\\ & \;\;=\frac{\left(\text{Nc}\times 50\times 50,000\right)-\left(\text{Ns}\times 50\times 10,000\right)}{\text{Nc}\times 50\times 50,000}\times 100. \end{array}$$

The bactericidal activity (%) of the inactivated serum for the mean cfu, Ni, was

(2) $$\frac{\left(\text{Nc}\times 50\times 50,000\right)-\left(\text{Ni}\times 50\times 50,000\right)}{\text{Nc}\times 50\times 50,000}\times 100.$$

### 2.8. Determination of Serum C3, C4, and IgG

Quantitative estimates of serum complement components C3 and C4 were performed using a TURBOX plus Analyzer, Orion Diagnostica, Finland. The method was based on the principle of immunoprecipitation reaction of a specific antibody with its antigen. The light scattering caused by antigen-antibody complexes was measured after incubation. The intensity of the scattered light was directly proportional to the concentration of the tested complement protein present in the serum sample. IgG was measured by immunonephelometry using DADE Behring reagents (USA) and an autoanalyzer. The procedure was according to the supplier's recommended protocol. The results were expressed in g/L.

### 2.9. Statistical Analysis

Data analyses were carried out using the Statistical Package for Social Sciences (SPSS). The methods used were independent *t-*test for comparison of two groups (arsenicosis patients and control subjects), Pearson correlations, and simple statistics. The results were considered significant when *P* was ≤0.05.

## 3. Results

### 3.1. Baseline Characteristics of the Study Subjects

The baseline characteristics showed the age of the patients varied from 14–65 yrs, and the mean body mass index (BMI) was 20.9 ± 3.5. In the control subjects, the age varied from 19 to 72 yrs, and the mean BMI was 22.4 ± 2.7. The whole population of patients had a mean monthly family income of about Bangladeshi Taka 5,000 (about USD 80) that varied from Taka 1,000–16,000. About one-third of the patients had no formal education; while 30% attended primary schools and the remaining had secondary school and higher education. The number of family members of the patients varied from 2 to 9 with a mean value of 5.0 ± 1.5. Of the unexposed subjects, only about 5% were illiterate, while the education levels of the rest were from primary to higher. The monthly family income of the controls varied from Taka 1,500–25,000 with a mean of about Taka 7,000 (USD 112). Their family members varied from 2 to 6.

### 3.2. Clinical Symptoms and Complications of Arsenic Toxicity

The clinical symptoms based on skin manifestations were recorded on questionnaire after interviewing and careful examination of the subjects. The symptoms included diffused and spotted melanosis with black and white appearances, rough and mottled skin, keratosis or hardening of the soles and palm with often formation of nodules and cracks. Spotted melanosis was more often seen on the throat, chest, back, or limbs. The duration of exposure has been found directly related to the severity of clinical disease. About one-third of the patients suffered from severe skin irritation including itchy rash. There was high prevalence of respiratory complications including asthma, bronchitis and cough. Long-term exposure to arsenic also caused conjunctivitis, disturbances in the peripheral vascular and nervous systems, and gangrene of the limbs. The patients had the mean duration of arsenic exposure for 7.4 ± 5.3 yrs that varied from 1 to 25 yrs with a median of 6.0 yrs.

### 3.3. Levels of Arsenic in the Drinking Water and Urine Samples

It was found that the drinking water of the patients contained 67–875 *μ*g/L of arsenic with the mean value of 216.2 ± 210.6 *μ*g/L and median of 156 *μ*g/L. The mean level of arsenic in the spot urine samples of the patients was 223.2 ± 302.4 *μ*g/L, while the median was 114.0 *μ*g/L and the maximum value was 1764 *μ*g/L. On the other hand, the mean arsenic concentrations in the drinking water and urine samples of the unexposed (control) populations were 11.3 and 29.4 *μ*g/L, respectively. Statistical analysis showed the levels of arsenic in the drinking water and urine samples of the patients were highly positively correlated (*P* < 0.001). 

### 3.4. Complement-Mediated Bactericidal Activity

**Figure 1:**
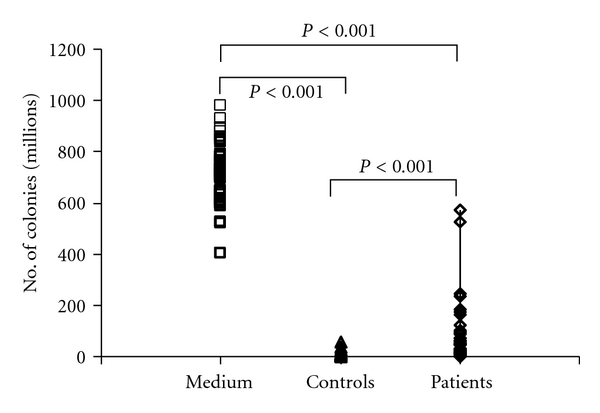
Number of *Escherichia coli* DH5*α* colonies (million cfu/mL) grown on agar plates from the bacterial cell suspension in PBS (Medium) and after the action of serum complements from healthy controls and arsenic patients. Before plating, the bacterial cell suspensions were diluted with PBS to 1 : 50,000 in Medium, and 1 : 10,000 both for serum samples from control subjects and arsenic patients. Compared with the Medium, complements from both the sera exhibited highly significant bacteriolytic effects (*P* < 0.001), while complements from arsenic patients showed significantly diminished bacteriolytic effects compared to the control subjects (*P* < 0.001).

It was found that the numbers of *Escherichia coli *DH5*α* colonies grown on agar plates without treating the bacterial cell suspensions with serum complement (PBS alone, negative control) varied from 401–978 × 10^6^ cfu/mL with a median of 714.5 × 10^6^ cfu/mL. On the other hand, the numbers of colonies formed after treating the BCS with serum complements from control healthy subjects varied from 1.1–54.8 × 10^6^ cfu/mL with a median of 4 × 10^6^ cfu/mL, and, after treating with serum complements from arsenic patients, the number of colonies varied from 1.5–570 × 10^6^ cfu/mL with a median of 47.3 × 10^6^ cfu/mL (Figure 1). Compared to the negative control experiments, complements from both healthy subjects and arsenic patients exhibited highly significant bacteriolytic effects (*P* < 0.001). However, complements from arsenic patients showed significantly diminished bacteriolytic effects (assessed by the large number of cfu) compared to the control subjects (*P* < 0.001). The bactericidal activity of complements from the arsenic patients varied widely from 24.6 to 99.9% compared to 92.2–100% in the control healthy subjects (Table 1). The complement-mediated bactericidal activity of the arsenic patients was significantly lower than that of the control subjects (*P* < 0.01). 

### 3.5. Effect of Complement-Inactivation on Bactericidal Activity

The bactericidal activity of the complement-inactivated serum from the control subjects varied from 6.2 to 35.3% whereas that from the arsenic patients varied from 3.3 to 33.0%. These results presented in Table 1 indicated that the complement-inactivated bactericidal activity of the arsenic patients was not significantly different from that of the control subjects.

### 3.6. Levels of Complement Component C3

It was found that, of the total arsenic patients, about 93% had normal levels of serum complement component C3 (normal level: 0.9–2.1 g/L) whereas 5% had above normal, and only 2% had below normal levels. On the other hand, 85% of the control subjects had normal levels, and the remaining 15% had above normal levels of serum complement C3. The mean level of C3 in the arsenic patients was 1.56 ± 0.36 g/L and that in the healthy control subjects was 1.68 ± 0.35 g/L. These results showed arsenic patients had lower levels of serum complement component C3 than the healthy subjects (Table 2).

### 3.7. Levels of Complement Component C4

The levels of complement component C4 in about 20% of the patients were found to be above normal. The remaining patients had serum C4 levels within the normal value (0.1–0.4 g/L). Among the control subjects, 90% had normal C4, 4% had above normal and the remaining 6% had below normal levels. The whole population of patients had the mean C4 level of 0.29 ± 0.11 g/L while that in the healthy control subjects was 0.25 ± 0.11 g/L. These data showed although the differences were not significant (*P* < 0.06), the complement component C4 levels in the arsenic patients were higher than that in the control subjects (Table 2).

### 3.8. Correlation of the Levels of C3 with Bactericidal Activity

**Figure 2:**
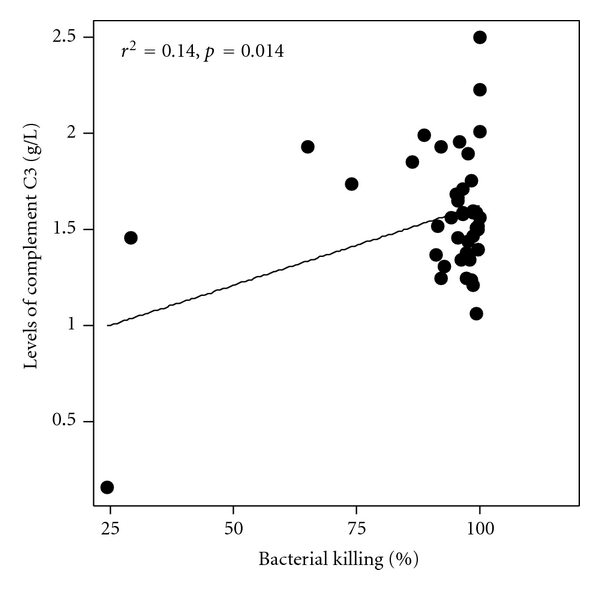
Correlation between the levels of serum complement C3 (g/L) from arsenicosis patients with bactericidal activity (bacterial killing, %) on *Escherichia coli* DH5*α* cells. The figure shows bacterial killing decreased as the levels of C3 decreased in arsenic toxicity, giving a direct correlation (*P* = 0.014).

It was found that about one-third of the total arsenic patients tended to have lower levels of C3; however, these values were around the lower limit of the normal range showing a mean value of 1.19 ± 0.42 g/L with corresponding bactericidal activity of 67.4 ± 20.2%. Analysis of the whole population data (*N* = 125) showed a significant direct correlation between the levels of C3 and complement-mediated bactericidal activity (*P* = 0.014) among the arsenicosis patients (Figure 2).

### 3.9. Levels of Serum IgG

To find out an explanation whether immunoglobulin G (IgG) was responsible for enhanced killing of bacteria after complement inactivation, IgG levels were measured in the fresh serum. It was found that arsenic patients had the mean serum IgG level of 24.3 ± 7.5 g/L compared to 13.8 ± 7.8 g/L in the healthy control subjects (normal level: 7.0–16.0 g/L). Statistical analysis showed IgG levels in the arsenicosis patients were significantly higher than that in the control subjects (*P* < 0.001). The results are shown in Table 2.

## 4. Discussions

Millions of people worldwide are chronically exposed to arsenic through drinking water, including an estimated 35–77 million people in Bangladesh [15]. In the present study, skin manifestations have been found as the prime and common features of arsenic toxicity that has been considered as definite exposure [2]. About 27% of the enrolled patients had BMI lower than 18.5 compared to only 6% of the control subjects. Thus, a relation between arsenic toxicity and poor nutritional status (low BMI) was confirmed in the present study, as described earlier [2]. It had been reported that infection could rapidly lead to nutritional stress and weight loss, thereby worsening nutritional status and immunologic function [16–18]. In a prospective cohort study on 14 patients with severe anorexia nervosa having BMI less than 14.0, serum complement C3 levels were found significantly lower than in the healthy controls [19]. We found a direct correlation, though marginally insignificant, between nutritional status (BMI) and serum complement protein C3 in the arsenicosis patients (*P* = 0.057). Poor nutritional status of the affected subjects might have a role in high retention of arsenic in the body.

Measurements of complement proteins C3 and C4 aid in the diagnosis of immunologic disorders, especially those associated with deficiencies of complement components. The proteins of complement system play important role in the innate immune response. The results of some clinical studies have suggested that complement activation exacerbated myocardial defect following ischaemic injury [20], has been involved in the generation of spontaneous atherosclerotic lesions, and may indeed be an initiating factor in lesion formation [21]. Assessment of the complement C3 and C4 concentration ratio (C3/C4 ratio) in serum has been suggested as a potential measurement to predict cardiovascular attacks [22]. New studies point to the complex interplay between the complement cascade and adaptive immune response, and complement is also being studied in association with ischemic injury as a target of therapy [9].

*Escherichia coli *DH5*α* used in this study is a nonpathogenic bacterium, and, therefore, no appreciable levels of specific antibody should be present in the sera of human subjects against this organism. Therefore, killing of bacteria would be solely due to serum complement. We found that the complement-mediated bactericidal activity in the patients was significantly lower than that in the control group. We also found the concentration of C3 in the arsenicosis patients was lower but that of C4 was higher, although none was significantly different than in the control subjects. However, about one-third of the arsenic patients had significantly lower values of C3 compared to the controls (*P* < 0.001). These sera containing lower levels of C3 showed highly significantly reduced bactericidal activity (67.4 ± 20.2%) compared to the healthy controls (99.1 ± 1.4%) and showed a value of *P* < 0.001.

The lower levels of C3 in the arsenic patients might be the reason for reduced complement function since a significant direct correlation was found between the levels of C3 and complement-mediated bactericidal activity (*P* = 0.014). The complement-inactivated bactericidal activity of the patients was similar to that of the control group. We reported earlier that the arsenic patients had significantly elevated levels of serum IgG, IgA, and IgE [5]. Since the arsenic patients suffer from melanosis, keratosis, cracks on foot soles and palms that might harbor bacterial infections, their sera might contain high levels of IgG antibodies against these organisms. These IgG antibodies might play a role in enhanced killing of bacterial cells, while the serum complements had been destroyed by heat inactivation. We reported similar findings in the pulmonary tuberculosis patients who had chronic lung infection [14].

The role of serum complement in immunotoxicity of heavy metals remains poorly understood. It had been reported in an old study that the lead workers had lower serum complement C3 and immunoglobulins IgG, IgM and IgA levels, as well as lower salivary IgA levels than the reference subjects [23]. In another study, the effects of gallium arsenide (GaAs) exposure as a single intratracheal dose on immunocompetence of B6C3F1 female mice were investigated and found that GaAs affected both humoral and cellular immune parameters in the mice and impaired the ability of the immune system to protect against B16F10 tumor challenge [24]. It had been found that bivalent nickel or cobalt at concentrations below 100 micro-M could stimulate the conversion of complement protein C3 to C3b faster than magnesium, which was the natural cofactor in the alternative pathway of complement activation, suggesting that the increased rate of C3-fragment production induced by nickel or cobalt ions was central for the immunotoxicity of these metals [25].

Deficiencies of complement proteins are frequently associated with an immunodeficiency state in which the patients suffer with recurrent bacterial infections from organisms that are normally susceptible to opsonization or lysis by complement. A recent study has shown low serum complement level in chronic liver disease significantly increases the risk of spontaneous bacterial peritonitis in children [26]. In patients with nonalcoholic liver cirrhosis, serum bactericidal activity had been found to be reduced and complement proteins C3 and C4 were found to have significantly low values [27]. In the arsenicosis patients, suppressed complement function might be a direct effect of chronic arsenic toxicity. It may be recalled that zymogen forms of complement protein C3 are activated both by the alternative and classical complement pathways, thereby making the activated fragment C3b takes part in bactericidal activity by either or both of these pathways. On the other hand, complement C4 takes part only in the classical pathway. Our finding of elevated levels of C4 in about 20% of the patients indicated underutilization of complement C4 and hence possibly suppressed activity of the classical complement pathway in drinking water arsenic toxicity.

## 5. Conclusion

Lower levels of serum complement protein C3 in the arsenicosis patients and reduced complement-mediated bactericidal activity reveal serum complement functions are suppressed in drinking water arsenic toxicity. The findings from this preliminary study show immunotoxicity of arsenic resulting impaired complement function that requires further investigation on a large population.

## Figures and Tables

**Table 1:** Complement-mediated and complement-inactivated bactericidal activities in the serum of arsenic patients and control subjects.

Study subjects	% Bactericidal activity (mean ± SD)	
	Complement-mediated	Complement inactivated
Arsenic patients *N* = 125	92.1 ± 16.0	19.4 ± 8.6
	Median: 97.3	Median: 18.6
	Range: 24.6–99.9	Range: 3.3–33.0
Control subjects *N* = 125	99.1 ± 1.4	18.2 ± 6.9
	Median: 99.4	Median: 17.1
	Range: 92.2–100	Range: 6.2–35.3
Statistics (*P* value)	<0.01	NS

**Table 2:** Levels of serum complements C3 and C4 and immunoglobulin G in the arsenic patients and control subjects.

Study subjects	Concentration in serum: (Mean ± SD) g/L		
	Complement C3 NV: 0.9–2.1	Complement C4 NV: 0.1–0.4	Immunoglobulin G NV: 7.0–16.0
Arsenic patients *N* = 125	1.56 ± 0.36	0.29 ± 0.11	24.3 ± 7.5
	Median: 1.56	Median: 0.27	Median: 22.7
	Range: 0.16–2.50	Range: 0.1–0.57	Range: 7.7–49.3
Control subjects *N* = 125	1.68 ± 0.35	0.25 ± 0.11	13.8 ± 7.8
	Median: 1.67	Median: 0.22	Median: 10.8
	Range: 0.9–2.64	Range: 0.05–0.49	Range: 4.2–31.6
Statistics (*P* value)	NS	NS (0.06)	<0.001

NV: normal value; NS: not significant.
